# Editorial: Differential diagnoses of thyroid neoplasms: Molecular and histological features and the impact on follow-up

**DOI:** 10.3389/fonc.2023.1125887

**Published:** 2023-01-31

**Authors:** Salvatore Sorrenti, Augusto Lauro, Daniele Pironi, Pietro Giorgio Calò, Salvatore Ulisse

**Affiliations:** ^1^ Department of Surgery, “Sapienza” University of Rome, Rome, Italy; ^2^ Department of Surgical Sciences, University of Cagliari, Monserrato, Cagliari, Italy

**Keywords:** thyroid cancer, molecular pathogenesis, diagnosis, ultrasound, staging, prognosis, therapy

Thyroid carcinoma (TC) is among the most frequent cancers in women (1–3). Its annual incidence increased over the last decades, mainly due to the improved ability to diagnose malignant transformation in small non-palpable thyroid nodules, to decline in more recent years in both sexes at a combined rate of 2.5% per year (1). Most of the epithelial TC are denoted as differentiated TC (DTC), including the papillary TC (PTC) and the follicular TC (FTC) histotypes, which, following dedifferentiation, are thought to give rise to the more aggressive poorly differentiated thyroid carcinoma (PDTC), and the incurable anaplastic TC (ATC) (4). Although derived from the same cell type, the different TC show specific histological features, biological activities and degree of differentiation due to different genetic alterations. Recently, multiple aspects of the clinical management of patients affected by thyroid nodules or thyroid malignancies, including diagnosis, treatment modalities and follow-up, are rapidly changing with the aim to resolve the still present clinical uncertainties (5–8).

Over the last years, a great advance in the comprehension of the molecular pathogenesis underlying TC progression, particularly in PTC (representing the most common thyroid malignancy), has led to a new classification of thyroid lesions into molecular subtypes with potential positive impact on the diagnostic accuracy of thyroid lesions, prediction of disease outcome, and patient’s tailored therapy (9–11). In addition, ultrasound (US) assessment of thyroid parenchyma has witnessed over the last decade a dramatic improvement with the introduction of new US software, such as contrast-enhanced US and US-elastography (USE), now recognized by the World Federation for Ultrasound in Medicine and Biology (WFUMB) and the European Federation of Societies for Ultrasound in Medicine and Biology (EFSUMB) as an essential part of thyroid nodule US examination (12–16). Moreover, the introduction of minimally invasive or remote-access surgical approaches for tumor ablation, design of small molecules inhibitors for the treatment of more aggressive TC, and a patient’s tailored follow up led to better disease outcomes and improved patient’s quality of life (17–21).

In the present issue, new insights into molecular pathogenesis, diagnosis, therapy and follow-up of TC patients are presented. Zhao et al. reported on the clinical significance of the co-stimulatory molecule B7-H3 expression in PTC. They showed that the level of B7-H3 could represent an independent biomarker for predicting lymph node metastases and disease recurrences in PTC patients, thus providing a new potential molecular parameter to refine risk-adapted therapeutic strategies, and also a putative novel drug target for patients affected by aggressive disease (22).

The autoimmune Hashimoto’s thyroiditis has been frequently shown to associate with DTC, but the liaison amongst these two clinical entities has yet to be elucidated. In this issue, Cappellacci et al. reported their single center experience on the incidence of Hashimoto thyroiditis in DTC patients, and assessed how this autoimmune disorder influences the risk of cancer recurrence (23).

The observation that both benign and malignant thyroid diseases (TD) frequently associate with extra-thyroidal malignancies has raised a considerable clinical interest (22–25). In particular, this relationship suggests: *i*) the need of an increased surveillance of TD patients at higher risk of developing extra-thyroidal malignancies; *ii*) the presence of common underlying molecular mechanism(s) responsible for these diseases, the comprehension of which could lead to a better and possibly personalized management of patients (22–25). In this context, Bellini et al. made a systematic review of the current evidence on the bidirectional relationship between thyroid and renal cancers. The authors showed that obesity and family history were the utmost common risk factors, and that genetic susceptibility was also present.

As above mentioned, USE is becoming an essential tool in the evaluation of thyroid nodules (12–16). Cantisani et al. performed a systematic review and a meta-analysis including 72 studies for a total of 13,505 patients and 14,015 thyroid nodules. In this study, the authors compared the diagnostic performances of qualitative, semi-quantitative and quantitative USE, and found that qualitative and semi-quantitative USE had very similar diagnostic accuracy, and that both of them were superior to the quantitative USE, with pooled sensitivity, specificity and AUC of 84%, 81%, and 0.89 respectively for qualitative USE, and 83%, 80%, and 0.93 for semi-quantitative USE. These data corroborate the valuable diagnostic role of USE in thyroid nodule evaluation, in accordance with the above reported guidelines from WFUMB and EFSUMB.

Although surgery is considered the gold standard in the treatment of papillary thyroid microcarcinoma (PTmC), active surveillance has become, over the last few years, an alternative option for PTmC patients. This approach was initially implemented in Japan to be then applied in other countries, but at present it has not yet been validated by the major Western Scientific Societies. In the work by Orlando et al. here reported, the results of nine studies published from 2017 to 2020 on this subject were analyzed. The authors concluded that, although data from western countries are still limited, active surveillance of PTmC appears to be a feasible strategy deserving further studies to confirm its usefulness in the clinical management of these patients. On the other hand, surgery is fundamental for the treatment of DTC and their more advanced forms (5). For the latter, however, no clear and specific guidelines have been drawn up to date. In the present issue, Bulfamante et al. retrospectively analyzed 111 patients with advanced DTCs, investigating the rate of radical excision, peri-procedural and post-procedural complications, quality of life, persistence, recurrence rates, and survival rates. The results were compared with those reported in the literature.

Among TC, ATC is a rare but highly aggressive and incurable form. Although some information on its molecular pathogenesis is available, little is known about risk factors. In their manuscript, Graceffa et al. reviewed the literature concerning risk factors for ATC, and examined analogous data from their own database. They found that ATC, in addition to being peculiar of elderly people, has a higher prevalence in individuals with a low level of education and a long history of multinodular goiter.

As previously stated, the increased comprehension of the genomic landscape of TC, and the possibility of identifying genetic alterations underlying more advanced diseases paved the way for a personalized therapy tailored on single patient characteristics. The last two manuscripts included in this special issue deal with the targeted therapies in patients with advanced DTC, PDTC and ATC (33, 34). In the first manuscript, Elia et al. describe a number of drugs approved by the Food and Drug Administration (FDA) for the therapy of the more aggressive and radioiodine (RAI)-resistant DTC and medullary thyroid cancer. In the second manuscript Macerola et al., besides providing a comprehensive review of the currently available targeted treatments for TC, describe the related predictive markers and testing methodologies.

In conclusions, the articles contained in the present special issue offer valuable information related to the clinical management of TC patients, which we hope will meet the interest of Frontiers in Oncology readers.

## Author contributions

All authors contributed to the initial draft of the manuscript, its revisions and all approved the final submitted version.
